# Estimated Costs and Cost-Effectiveness of a Pediatric Weight Management Program

**DOI:** 10.1001/jamanetworkopen.2025.10087

**Published:** 2025-05-14

**Authors:** Meg Simione, Sophie Wagner, Caroline J. Kistin, Kelsey A. Egan, Sheila Kelly, William G. Adams, Elsie M. Taveras, Chin Hur

**Affiliations:** 1 Department of Pediatrics, Mass General for Children, Boston, Massachusetts; 2 Department of Pediatrics, Harvard Medical School, Boston, Massachusetts; 3 Department of Medicine, Columbia University Irving Medical Center, New York City, New York; 4 Hassenfeld Child Health Innovation Institute, Department of Health Services, Policy, and Practice, Brown University School of Public Health, Providence, Rhode Island; 5 Division of Health Services Research, Department of Pediatrics, Boston Medical Center, Boston University Chobanian and Avedisian School of Medicine, Boston, Massachusetts

## Abstract

**Importance:**

Childhood obesity remains a significant public health challenge, with ongoing racial, ethnic, and socioeconomic disparities in its prevention and treatment. Economic evaluations of pediatric obesity interventions are essential for guiding resource allocation in health care settings.

**Objective:**

To develop and analyze a simulation model of the Connect for Health program to estimate the costs of implementing the program and assess its cost-effectiveness.

**Design, Setting, and Participants:**

This economic evaluation used a Markov cohort model based on the Connect for Health randomized clinical trial (RCT) conducted from June 2014 through March 2016 and the Connect for Health implementation study conducted at 2 academic medical centers in Boston, MA. The study first estimated costs of implementation for new sites, and then simulated program implementation in a cohort that mirrored the RCT population. Data were analyzed from October 2023 to March 2025.

**Exposure:**

The Connect for Health pediatric weight management program.

**Main Outcomes and Measures:**

Outcomes include costs associated with program implementation and the incremental cost-effectiveness ratio (ICER) per quality-adjusted life-year (QALY) gained.

**Results:**

This study used a cohort of 2494 individuals to mirror the RCT population and included 1178 female participants (47%), a mean (SD) age of 8.0 (3.0) years, and a mean (SD) body mass index *z* score of 1.91 (0.56). The costs of implementing the Connect for Health program in primary care settings were estimated at $58 000 in 1-time startup costs and $1300 in ongoing monthly costs. The economic model showed that the Connect for Health strategy had an ICER of $10 554 per QALY gained over 2 years compared with no intervention. The study found a threshold of 534 children in a cohort was needed to effectively amortize costs.

**Conclusions and Relevance:**

In this economic evaluation of a pediatric weight management program, the Connect for Health program was estimated to be cost-effective. These findings are important for health care systems that serve children who are less likely to receive obesity-related care in making decisions about program adoption.

## Key Points

**Question:**

What are the estimated costs and cost-effectiveness of implementing Connect for Health, a systems-level pediatric weight management program, in primary care settings?

**Findings:**

In this economic evaluation of 2494 individuals, the Connect for Health program was associated with a cost-effectiveness ratio of $10 554 per quality-adjusted life-year gained over a 2-year implementation period.

**Meaning:**

These findings suggest that the Connect for Health program is a potentially cost-effective strategy for managing childhood obesity in primary care settings, particularly when implemented at scale.

## Teaser

This economic evaluation examines a simulated model of the Connect for Health program to estimate the costs of implementing the program and assessing its cost-effectiveness.

## Introduction

Childhood obesity continues to be highly prevalent, with persistent racial, ethnic, and socioeconomic disparities despite ongoing national efforts at prevention and treatment.^1,2,3,4^ Prevention and treatment programs are needed in the pediatric primary care setting due to the lack of options available for addressing obesity among children,^5^ particularly those who are from low-income families and from minoritized racial and ethnic groups who are less likely to receive obesity-related care.^6^ Studying program implementation is important not only to understand how to promote uptake in primary care but also to understand the costs of implementing programs and program cost-effectiveness. Economic evaluations provide decision-makers with complete information that allows them to assess the true costs of implementation, helping them make informed investment decisions, especially given limited resources.^7,8,9^ This understanding can facilitate the spread, equitable uptake, and sustainment of effective interventions for children at high risk for obesity and its complications.

Previous studies have emphasized the importance of preventative programs and sustained management to mitigate the long-term consequences of obesity. However, few studies have examined the costs of implementing childhood obesity interventions despite the critical importance of the information to ensuring all children, particularly children who are from low-income families and from racial and ethnic minoritized groups, have access to the care they require.^10,11,12,13,14^ Additionally, studies that have examined cost-effectiveness often focus on the costs related to the intervention and exclude the costs required to implement and maintain a program.^9,15,16,17,18^

We conducted an economic evaluation of Connect for Health, an evidence-based, systems-level pediatric weight management program for primary care, which was specifically designed to support children from low-income families and from racially and ethnically minoritized groups who are disproportionately affected by obesity.^19,20^ The purpose of the study was to develop and analyze a simulation model of the Connect for Health program to estimate the costs of implementing the program and assess its cost-effectiveness.

## Methods

This economic evaluation was approved by the institutional review board at Mass General Brigham. We followed the Consolidated Health Economic Evaluation Reporting Standards (CHEERS) reporting guideline^23^ to conduct the current study and present the results of our analysis. Informed consent was not required because it was deidentified data of an existing dataset.

Connect for Health is a pediatric weight management program for children ages 2 to 12 years that leverages clinical and community resources by guiding primary care clinicians in screening and best management practices through clinical decision support tools, providing families with educational materials to support behavior change, and providing families with information about resources in their community (ie, community resource guide). The 1-year randomized clinical trial (RCT) of Connect for Health examined the comparative effectiveness of enhanced primary care (clinical decision support tools, family educational materials, and a text-messaging program) vs enhanced primary care plus individualized health coaching.^19,20^ Both groups of the RCT improved child BMI and quality of life.^20^ Following the RCT, the program was adapted for dissemination to organizations that serve low-income children and children from racially and ethnically minoritized groups,^21^ and a study was conducted to evaluate strategies to promote the uptake of Connect for Health.^22^ The implementation strategies included conducting clinician training; providing technical assistance, practice facilitation, and feedback to program users; creating a virtual learning community; and aligning the program with the health care system’s performance metrics. Detailed cost data about implementation for this study were collected at pediatric primary care practices and community health centers at Boston Medical Center (BMC) and Massachusetts General Hospital (MGH), 2 health care systems that serve the Greater Boston, Massachusetts, area. The study protocols that include detailed descriptions of the intervention, the RCT and implementation study, and implementation strategies have previously been reported.^19,22^

### Costing Methodology

We estimated the costs of Connect for Health from a health system perspective with the goal of providing critical information for other health care systems considering and planning implementation of the program. With the objective of making the costs of our 2 sites more generalizable to the rest of the nation, we adopted a time-driven activity-based costing (TDABC), also called microcosting, approach to estimate costs associated with program activities. This approach involves delineating activities undertaken and quantifying personnel time, wages, and event frequency.^24^ In addition to TDABC of personnel resources, we identified nonpersonnel costs associated with supplies and services needed for the program. Costs were systematically gathered and categorized into their respective predefined phase of the program rollout, including preimplementation, implementation, and maintenance. The preimplementation phase was dedicated to planning and stakeholder engagement, the program launch marked the implementation period, and maintenance began when we gradually reduced grant funding.^22^

We calculated personnel time based on hourly estimates collected during the preimplementation and implementation phases of the program. Time estimates were compiled through review of program records, administrative documents, and calendar data where applicable. Personnel times were aggregated for each activity and multiplied by national hourly wage estimates from the Bureau of Labor Statistics’ (2023) Occupational Employment Statistics according to an individual’s position title and degree.^25^ A standard fringe rate of 32% was applied to all wages. Nonlabor costs for supplies and text messaging operations were based on actual expenditures tracked through receipts and payment invoices.

We gathered cost estimates from both sites (eTable 1 in Supplement 1) and then used them to calculate the anticipated expenses that future implementation sites are likely to incur (Table 1 and eTable 2 in Supplement 1). The coordinating center, MGH, had costs for developmental activities to prepare the program for implementation which would not be expected of future sites; thus, we excluded them from our analysis (eTable 3 in Supplement 1). Costs that would be incurred during the maintenance phase were determined from data and expert consensus across multiple implementation sites. All cost calculations were made in 2025 US dollars.

**Table 1.** Estimated Costs of Connect for Health Program for Markov Model Analysis

Phase and category	Cost, $^a^	SA Range, $
Preimplementation^b^	45 825	22 913-68 738
Text messaging program and execution fees	18 502	9251-27 753
Design and build EHR tools	11 777	5888-17 665
Identify and train implementation team members	7549	3775-11 324
Conduct clinician interviews and caregiver surveys to understand program and implementation needs	6121	3061-9182
Conduct meetings about program implementation to gain buy-in	721	360-1081
Perform clinical environment and workflow audits to prepare for implementation	594	297-892
Educate, inform, and train clinicians on the intervention	561	280-841
Implementation^b^	21 960	10 980-32 939
Educate, inform, and train clinicians on the intervention	9546	4773-14 319
Survey families regarding experience of care	5589	2795-8384
Monitor usage of EHR tools	3504	1752-5256
Align program with quality improvement initiative	2388	1194-3581
Provide program feedback to clinicians about their use of EHR tools	932	466-1398
Maintenance^c^	18 340	9170-27 511
Annual operating expenses for text messaging program	13 154	6577-19 731
Conduct ongoing clinician training, practice facilitation, and technical assistance	2411	1205-3616
Provide program feedback to clinicians about their use of EHR tools	1550	775-2326
Monitor usage of EHR tools	429	215-644
Adapt EHR tools as needed	796	398-1194

Abbreviations: EHR, electronic health record; SA, sensitivity analysis.

^a^ All costs presented in 2025 USD.

^b^ Preimplementation and implementation activities are considered 1-time expense, accounted for at the start of the Markov Model analysis.

^c^ Maintenance activities are ongoing estimated expenses that the health care organization would expect to spend. They are applied monthly throughout the Markov Model analysis.

#### Sensitivity Analysis

To account for uncertainty of our cost estimates, we conducted sensitivity analyses, which were performed within the broader cost-effectiveness evaluation using a Markov model detailed in the following section. In the sensitivity analysis, we established a lower cost boundary for the intervention by considering scenarios where institutions already had electronic health record (EHR) best practice alerts in place, reducing the need for additional resources. We varied all other costs by 50% more or less of their estimated value. These adjustments allow us to better understand the impact of cost uncertainties on the overall economic evaluation of the program.

### Cost-Effectiveness Analysis

We developed a Markov cohort model to evaluate the economic and clinical outcomes of the Connect for Health RCT compared with a no-intervention standard care scenario. The model operated over 2 years, reflecting the follow-up period of the Connect for Health RCT,^20,22,26^ which provided the necessary data for our analysis. The model simulated the BMI *z* score trajectories of children with baseline characteristics from the enhanced primary care arm in the RCT (Table 2).^22,26^ The model compares 2 strategies: (1) no intervention and (2) Connect for Health. Each month in the model, individuals in either strategy could either maintain their current BMI *z* score, reduce their BMI *z* score, or die from all-cause mortality (eFigure in Supplement 1). We derived a natural BMI trajectory estimating BMI change without treatment from the US Centers for Disease Control and Prevention’s extended BMI-for-age growth charts.^27^ In the no intervention strategy, we assumed no treatment was received, and no change in BMI *z* score occurred, following approaches used in prior studies.^28^ BMI change *z* score changes for patients in the Connect for Health strategy were modeled from those observed in the enhanced primary care group of the Connect for Health RCT at 1 and 2 years.^20,26^

**Table 2.** Input Parameters for 2-Year Markov Model Analysis of Connect for Health Program

Parameter	Estimate (SE)	Distribution^a^	Source
BMI *z* score changes over time			
Baseline	1.91 (0.53)	NA	Taveras et al,^20^ 2017
1 y	−0.04 (0.04)	β	Taveras et al,^20^ 2017
2 y	−0.06 (0.07)	β	Taveras et al,^26^ 2018
Utilities			
Initial utility	0.80 (0.02)	NA	Keating et al,^30^ 2011
1 Unit of BMI *z* score decrease	0.04 (0.02)	β	Bairdain and Samnaliev,^20^ 2015
Costs^b^			
Program initial cost	$67 784 ($33 892)	γ	Methods
Program continuing costs	$1528 ($764)	γ	Methods

Abbreviations: BMI, body mass index; NA, not applicable.

^a^ The parameter was not varied in 1-way or probabilistic sensitivity analyses and was kept as its base case value.

^b^ Costs were varied by ±50% for sensitivity analysis range. Since no empirical standard error was available, values in parentheses represent half of the mean as a reference for the sensitivity analysis range.

An increase in utility was incorporated based on a reduction in BMI *z* score, reflecting the reported association between BMI and health-related quality of life.^29^ Initial quality of life utility values for individuals with a BMI at or above the 85th percentile were sourced from published literature.^29^ We categorized the estimated costs of Connect for Health as 1-time or continuing and applied them at the model’s start and monthly, respectively, in the Connect for Health strategy, while the no intervention was assumed to have no costs (Table 1). The model cohort consisted of 2494 eligible children (with BMI in the 85th percentile or higher), based on baseline estimates at BMC,^22^ which also served as the primary reference for our cost assessments. We applied a discount rate of 3% for future costs and utilities.^30^

### Statistical Analysis

Our primary endpoints included quality-adjusted life-years (QALYs), total costs per patient treated in 2025 US dollars, and incremental cost-effectiveness ratios (ICERs). An ICER is the ratio of the increase in costs to the increase in QALYs between 2 strategies; in this analysis, no intervention serves as the reference strategy. A strategy is deemed cost effective if the ICER is below the willingness-to-pay (WTP) threshold of $100 000 per QALY.^31^ The preferred strategy maximizes QALYs while maintaining an ICER below $100 000 per QALY. Secondary end points examined the mean change in BMI *z* scores from baseline.

#### Sensitivity Analysis

We conducted 1-way and probabilistic sensitivity analyses to account for uncertainty in model inputs and to validate model outcomes. In 1-way sensitivity analysis, key parameters vary 1 at a time while all others retain their base-case value. Ranges for BMI *z* score changes were derived from the Connect for Health RCT,^20,26^ while ranges for utilities were derived from the literature.^29,32,33^ Probabilistic sensitivity analyses involved simulating the model multiple times, drawing parameter values from γ distributions for costs and β distributions for clinical and utility parameters. We determined the percentage of times each strategy was preferred over a range of WTP thresholds. All analyses were performed using Python software version 3.9.19 (Python) from October 2023 to March 2025.

## Results

### Costing Results

The costing results of the preimplementation, implementation, and maintenance phases are shown in Table 2. Preimplementation costs totaled approximately $45 825 (±50% sensitivity analysis range [SA range]: $22 913-$68 738), covering essential work that occurred in preparation for the program’s implementation. Costs included setting up the text messaging infrastructure, developing the EHR best practice alerts system, and training the implementation team. The most significant expense during this phase was the infrastructure and execution fees for the text messaging program, which were estimated at $18 502 (SA range: $9251-$27 753) for a single site.

Implementation costs were estimated at $21 960 (SA range: $10 980-$32 939), with ongoing education and training of clinicians being a primary expense. Costs were also incurred for surveys to get feedback from families on their experiences with the clinical services, which are crucial for evaluating program impact and aligning with quality improvement initiatives. Maintenance costs, required for the ongoing operation and updating of the program, reached $18 340 (SA range: $9170-$27 511) annually. This phase included costs for the regular operation of the text messaging program and continuous training and technical assistance, ensuring sustained program effectiveness and adaptability.

### Cost-Effectiveness Results

The model reproduced the baseline population and BMI distribution of the Connect for Health RCT, simulating 2494 children at a mean (SD) age of 8 (3.0) years with a starting mean (SD) BMI *z* score of 1.91 (0.56), of whom 1178 were female (47%). The model replicated the relative change in BMI *z* score from baseline, reflecting the intention-to-treat values from the enhanced primary care strategy of the Connect for Health RCT. In the base case, the Connect for Health strategy resulted in a relative BMI *z* score change from baseline of 0.10 after 2 years. The strategy led to an incremental gain of 0.0039 QALYs compared with no intervention. The Connect for Health strategy costs $40.87 per individual enrolled in the program. The ICER was $10 554 per QALY gained, indicating the cost-effectiveness of the intervention under the WTP threshold of $100 000 per QALY (Table 3).

**Table 3.** Cost-Effectiveness Results of Connect for Health Program Over 2 Years

Strategy	Cost	Incremental cost	Effectiveness, QALY	Incremental effectiveness, QALY	ICER, $/QALY gained
No intervention	$0.00	NA	1.6204	NA	NA
Connect for Health	$40.87	$40.87	1.6243	0.0039	$10 553.68

Abbreviations: ICER, incremental cost-effectiveness ratio; NA, not applicable; QALY, quality-adjusted life-year.

#### Sensitivity Analysis

Uncertainty in model inputs and their impact on results was explored through both 1-way and probabilistic sensitivity analyses. One-way sensitivity analyses found that the model’s outcomes were most sensitive to changes in the number of patients in the program’s population and quality of life utility value associated with BMI *z* score reduction (Figure). When the population size was reduced to below 534 individuals, the ICER exceeded the WTP threshold. This suggests that the economic viability of the Connect for Health strategy relies on maintaining sufficient enrollment levels, effectively amortizing substantial startup costs across a larger number of participants to minimize cost per patient. Notably, despite the uncertainty in quality-of-life utility values associated with BMI changes in children, our analysis indicated that the strategy was cost-effective with a utility increase of only 0.009 for a full unit reduction in BMI *z* score. This finding supports the program’s effectiveness amid variability in these estimates. Probabilistic sensitivity analysis, which incorporated variability in all model parameters simultaneously, further confirmed the stability of the model results. It found that in 85% of 10 000 iterations, the Connect for Health strategy remained the preferred option under the WTP threshold of $100 000 per QALY at 2 years.

**Figure.**
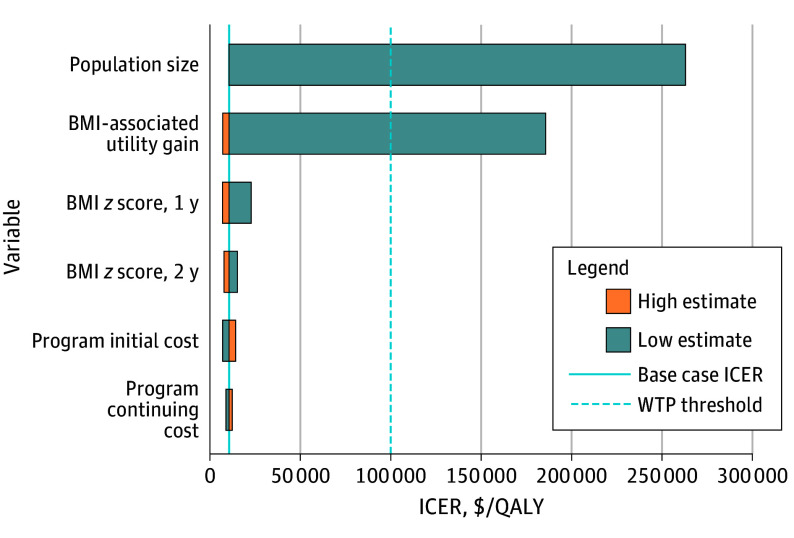
Connect for Health Weight Management Program 1-Way Sensitivity Analysis Results, No Intervention vs Connect for Health When patient population size decreases to fewer than 534 participants in the Connect for Health program, the incremental cost-effectiveness ratio (ICER) exceeds the willingness-to-pay (WTP) threshold, rendering the program not cost-effective when all other parameters remain constant. ICERs were $263 209 for population size, $185 745 for body mass index (BMI)–associated utility gain, $22 824 for BMI *z* score change at 1 year, $15 181 for BMI *z* score change at 2 years, $14 063 for program initial cost, and $12 321 for program continuing cost. The expected value is $10 554. QALY indicates quality-adjusted life-year.

## Discussion

In this economic evaluation, we assessed the implementation costs and cost-effectiveness of Connect for Health, a systems-level pediatric weight management program, over 2 years compared with no intervention, with the primary goal of informing decisions about implementing similar programs in health care systems across the US. Distinct from other cost-effectiveness studies, our methodology leveraged implementation costs in the clinical setting to project expenses at future sites. We estimated the total startup cost to be approximately $68 000 with ongoing monthly costs of $1500. The study found a modest incremental gain in QALYs per individual but achieved a favorable ICER of $10 554 per QALY, well below the established willingness-to-pay threshold of $100 000 per QALY, largely because the costs, when spread over the patient population, were not very high. These findings underscore the dependency of the program’s economic viability on a sufficient scale, highlighting the need to maintain a minimum population size to effectively distribute startup costs. The sensitivity analyses were robust to input variations, such as utilities, cost, and BMI change. Given the substantial margin under the WTP threshold, we can infer that the program is likely to remain cost-effective across various health care settings despite differences in resource availability, patient demographics, and existing health care infrastructure.

Connect for Health supports clinicians in screening and best management practices and provides family education for behavior change. In a clinical trial, it was found to reduce BMI *z* score,^20^ demonstrating that results can be achieved with minimal resources at a systems level. Cost-effectiveness studies have been conducted on intensive health behavior and lifestyle treatment programs that meet the 26-contact-hour requirements recommended by the American Academy of Pediatrics (AAP) and the US Preventive Services Task Force (USPSTF).^34,35,36^ These studies have reported positive child BMI outcomes but require more resources than Connect for Health and are, therefore, more expensive.^15,37^ Additionally, to date, few studies have reported the costs of implementation or maintenance and have considered this when calculating cost-effectiveness.^9,15,16,17^ By incorporating implementation costs, we have represented the program’s true costs, which allows health care systems to make informed decisions when adopting a program, leading to program sustainability.^7,8,9^

Our system-level approach to providing obesity-related care has shown that even lower-cost interventions can be an effective adjunct in managing childhood obesity. Connect for Health aligns with the AAP’s clinical practice guidelines and USPSTF’s recommendations^36,38^ in providing screening, evaluating medical comorbidities, and behavior change support as a necessary complement to high-intensity programs to reduce racial, ethnic, and socioeconomic disparities. The program ensures all children are identified, screened, and receive the care they require, highlighting the potential for less intensive, more affordable interventions to deliver meaningful results in childhood obesity management. Given the absence of programs in pediatric primary care, a system-level, cost-effective program is critically needed.

The Connect for Health Program offers a viable model for health care systems—particularly serving children from low-income communities who have historically not received adequate obesity-related care—to integrate effective pediatric weight management programs. Fifty-seven percent of federally qualified health centers that receive federal funding would have a sufficient patient cohort size (over 534 children with overweight or obesity) to implement the program cost-effectively and would reach approximately 1.85 million children (eMethods in Supplement 1).^39,40,41^ This finding underscores the program’s broad feasibility and potential impact on reducing pediatric obesity rates and improving health outcomes for children. Policymakers and health care organizations should recognize the program’s relevance and prioritize sustained funding for such programs that provide care at a systems level. Providing necessary care and education to children now will have implications and cost savings for Medicaid and health care systems in the future. Furthermore, investing in future sites and studying long-term impacts can provide additional validation of the program’s cost effectiveness and inform broader policy decisions.

### Limitations

This study has limitations. Our cost estimates were primarily derived from 2 health care systems in the northeastern US, which may not reflect cost structures in different geographic locations or health care settings. Moreover, our estimates for the cost and effect of intervention were based on a single trial and follow-up cohort, potentially limiting generalizability. However, probabilistic sensitivity analyses allowed us to generate uncertainty intervals around the model estimates and final conclusions. Furthermore, the analysis was constrained by the 2-year follow-up data from the Connect for Health RCT, limiting our ability to evaluate long-term outcomes and cost-effectiveness. Additionally, while we assumed no BMI z-score change in the absence of treatment, BMI can fluctuate in the clinical setting due to a myriad of reasons. Future research incorporating longer-term observational data may help refine this assumption. The impact of extending the program’s time horizon was not explored due to limited follow-up data, as extrapolating beyond 2 years would have required strong assumptions about long-term BMI trajectories. Finally, the findings indicate significant sensitivity to population size, highlighting potential challenges in smaller-scale implementations or settings with fluctuating patient volumes.

## Conclusions

The findings of this economic evaluation suggest that the Connect for Health program demonstrated economic viability and effectiveness in managing childhood obesity. Policymakers and health care clinicians should recognize the program’s relevance and prioritize sustained funding for pediatric weight management programs that screen and provide clinician guidance and family education at a systems level. Investing in future sites and studying long-term impacts can further validate the program’s cost-effectiveness and inform broader policy decisions.

## Notes

Supplement 1. **eTable 1.** Site-based Costs of the Connect for Health Weight Management Program at Massachusetts General Hospital (MGH) and Boston Medical Center (BMC) Over the Preimplementation and Implementation Periods **eTable 2.** Detailed Cost Estimates Connect for Health Program for Markov Model Analysis **eTable 3.** Coordinating Center Costs to Adapt the Connect for Health Weight Management Program for Implementation **eFigure. **Simplified Model Schematic for Connect for Health Markov Model Analysis **eMethods. **Estimation of Overweight and Obesity Prevalence Among Children Aged 2 to 12 Years Served in Federally Qualified Health Centers
eReferences.

Supplement 2.
Data Sharing Statement
