# Supplementary material for: Estimated Costs and Cost-Effectiveness of a Pediatric Weight Management Program
Source: JAMA Netw Open. 2025 May 14;8(5):e2510087. doi: 10.1001/jamanetworkopen.2025.10087 (PMC12079289; doi:10.1001/jamanetworkopen.2025.10087)
Supplement: Supplement 1. — eTable 1. Site-based Costs of the Connect for Health Weight Management Program at Massachusetts General Hospital (MGH) and Boston Medical Center (BMC) Over the Preimplementation and Implementation Periods eTable 2. Detailed Cost Estimates Connect for Health Program for Markov Model Analysis eTable 3. Coordinating Center Costs to Adapt the Connect for Health Weight Management Program for Implementation eFigure. Simplified Model Schematic for Connect for Health Markov Model Analysis eMethods. Estimation of Overweight and Obesity Prevalence Among Children Aged 2 to 12 Years Served in Federally Qualified Health Centers eReferences. [file jamanetwopen-e2510087-s001.pdf]

Supplemental Online Content

Simione M, Wagner S, Kistin CJ, et al. Estimated costs and cost-effectiveness of a pediatric weight management program. *JAMA Netw Open*. 2025;8(5):e2510087.
doi:10.1001/jamanetworkopen.2025.10087

eTable 1. Site-based Costs of the Connect for Health Weight Management Program at Massachusetts General Hospital (MGH) and Boston Medical Center (BMC) Over the Preimplementation and Implementation Periods

eTable 2. Detailed Cost Estimates Connect for Health Program for Markov Model Analysis

eTable 3. Coordinating Center Costs to Adapt the Connect for Health Weight Management Program for Implementation

eFigure. Simplified Model Schematic for Connect for Health Markov Model Analysis

eMethods. Estimation of Overweight and Obesity Prevalence Among Children Aged 2 to 12 Years Served in Federally Qualified Health Centers

eReferences.

This supplemental material has been provided by the authors to give readers additional information about their work.

© 2025 Simione M et al. *JAMA Network Open*.

eTable 1. Site-based Costs of the Connect for Health Weight Management Program at Massachusetts General Hospital (MGH) and Boston Medical Center (BMC) Over the Pre-implementation and Implementation Periods.

Phase and Category	MGH	BMC
Preimplementation	$44,200	$21,146
Text messaging program startup and execution fees	$18,502	-
Design and build electronic health record (EHR) tools	$7,293	$11,687
Identify and train implementation team members	$1,634	$7,492
Conduct clinician interviews and caregiver surveys to understand program and implementation needs	$14,564	$1,609
Conduct meetings about the program implementation to gain buy-in	$1,060	$358
Perform clinical environment and workflow audits to prepare for implementation	$590	-
Educate, inform, and train clinicians on the intervention	$556	-
Implementation[a]	$89,875	$17,273
Text messaging program operating expenses	$63,576	-
Survey families regarding experience of care	$12,826	$4,578
Monitor usage of EHR tools	$5,414	$5,890
Educate, inform, and train clinicians on the intervention	$4,765	$6,806
Align program with quality improvement bonus	$2,369	-
Provide program feedback to clinicians about their use of EHR tools	$925	-
**Total**	**$134,075**	**$38,419**

Note. All costs are reported in 2025 U.S. dollars. Inflation adjustments were applied using the Consumer Price Index where applicable.

[a] Implementation costs reflect expenses over the 28-month and 34-month implementation periods for MGH and BMC, respectively.

© 2025 Simione M et al. *JAMA Network Open*.

eTable 2. Detailed Cost Estimates Connect for Health Program for Markov Model Analysis.

Phase, Category, and Activity	Personnel involved	Item Cost	Total
**Preimplementation[a]**			**$45,825**
Text messaging program and execution fees			$18,502
Start-up purchase of texting service	N/A	$7,455	
Execution fee for texting service	N/A	$11,047	
Design and build electronic health record (EHR) tools			$11,777
Obtain approval of EHR Integration	Site-PI, PD	$846	
IT and EHR/BPA planning	CC, Co-I, Site-PI	$3,890	
Connect for Health IT planning	IT/Epic, PD, PM	$323	
Internal SmartSet discussion	IT/Epic, Site-PI	$124	
EHR build and text messaging referral integration	Co-I, IT/Epic, Site-PI	$5,733	
Text messaging service onboarding	PD, Site-PI	$249	
Text messaging service-hospital system sync	PD, Site-PI	$125	
C4H launch preparation	CC, Co-I, Site-PI	$486	
Identify and train implementation team members			$7,549
Select Clinician Champion	Site-PI	$700	
Train and support clinicians	CC, Site-PI	$6,850	
Conduct clinician interviews and caregiver surveys to understand program and implementation needs			$6,121
Clinician interviews			*$1,902*
Interview clinicians	CC, Site-PI	$1,245	
Provide clinician incentives	N/A	$373	
Analyze clinician interviews	CRC, IT/Epic, PD	$284	
Parent stakeholder surveys			*$4,219*
Conduct parent surveys	CRC, IT/Epic, PD	$1,551	
Provide parent survey incentives	N/A	$949	
Analyze parent surveys	DA/Bio, PD, Site-PI	$1,720	
Conduct meetings about program implementation to gain buy-in			$721
Conduct program implementation meetings	CRC, PD, PM, Site-PI	$721	
Perform clinical environment and workflow audits to prepare for implementation			$594
Audit clinical workflow	HC	$46	
Observe clinic workflows	CC, HC	$548	
Educate, inform, and train clinicians on the intervention			$561
Train clinicians on childhood obesity and C4H intervention	CRC, CC, HC	$561	
**Implementation[a]**			**$21,960**
Educate, inform, and train clinicians on the intervention			$9,546
Introduce tool and provide local technical assistance	CRC, CC, PC	$2,689	
Initial training of clinicians on BPA and SmartSet tools	CC, Site-PI	$1,245	
Educate resident physicians on SmartSet	CC	$700	
Conduct hands-on training with clinicians	CC	$4,913	

© 2025 Simione M et al. *JAMA Network Open*.

Phase, Category, and Activity	Personnel involved	Item Cost	Total
Survey families regarding experience of care			$5,589
Conduct family experience of care survey	CRC	$3,101	
Provide survey incentives	N/A	$1,628	
Analyze survey responses	DA/Bio, PD, Site-PI	$860	
Monitor usage of EHR tools			$3,504
Track EHR usage metrics	CRC, IT/Epic	$1,378	
Define EHR success indicators	CRC, CC, PC, PD, PM	$368	
Update EHR/BPA as needed	CRC, CC, IT/Epic, PC, PD, PM	$1,758	
Align program with quality improvement initiative			$2,388
QI bonus onboard meeting	CC, Co-I, PC, PD, PM	$2,007	
Initiate QI bonus alignment	CC, Co-I, PM	$381	
Provide program feedback to clinicians about their use of EHR tools			$932
Generate EHR usage reports	CRC	$600	
Share EHR usage reports	CRC, PM	$333	
**Maintenance[b]**			**$18,340**
Annual operating expenses for text messaging program			$13,154
Ongoing licensing and support	N/A	$13,154	
Conduct ongoing clinician training, practice facilitation, and technical assistance			$2,411
Continuous training and encouragement	CC	$2,411	
Provide program feedback to clinicians about their use of EHR tools			$1,550
Audit and share feedback reports with teams	CC, PM	$1,550	
Monitor usage of EHR tools			$429
Track ongoing EHR performance	CRC, IT/Epic	$429	
Adapt EHR tools as needed			$796
Adapt EHR tools as needed	CC, IT/Epic	$796	

Note. *Abbreviations:* IT, information technology; BPA, Best Practice Alert; EHR, electronic health records; CC, clinician champion; CRC, clinical research coordinator; Co-I, Co-Investigator; IT/Epic, IT or Epic analyst; DA/Bio, data analyst or biostatistician; HC, health coach; N/A, not applicable; PC, practice coach, PD, project director; PM, project manager; Site-PI, Site Principal Investigator. All costs are reported in 2025 U.S. dollars. Inflation adjustments were applied using the Consumer Price Index where applicable.

[a] Preimplementation and implementation activities are considered one-time expense, accounted for at the start of the Markov Model analysis.

[b] Maintenance activities are ongoing estimated expenses that the healthcare organization would expect to spend. They are applied monthly throughout the Markov Model analysis.

© 2025 Simione M et al. *JAMA Network Open*.

eTable 3. Coordinating Center Costs to Adapt the Connect for Health Weight Management Program for Implementation.

Category	Cost
Design and build of electronic health record (EHR) tools	$20,540
Create virtual learning community (VLC) for clinicians	$14,732
Develop clinician interview guide and parent surveys to understand programs needs and implementation	$10,935
Create new multimodal patient education materials (i.e., video)	$8,240
Adapt existing family educational materials and community resource guide	$6,330
Develop and maintain program website	$5,935
Develop family experience of care survey	$3,918
Refine text message library	$3,620
Create materials for clinician training and education	$3,058
Conduct stakeholder engagement for telehealth adaptation[a]	$1,479
COVID-19 adaptations to materials[a]	$835
**Total**	**$79,622**

Note. All costs listed are unique to Massachusetts General Hospital (MGH), the coordinating center, for the development and maintenance of the Connect for Health Program. No other participating sites will incur these costs. All costs are reported in 2025 U.S. dollars. Inflation adjustments were applied using the Consumer Price Index where applicable.

[a] In response to the COVID-19 pandemic, modifications were implemented to facilitate telehealth and remote engagement.

© 2025 Simione M et al. *JAMA Network Open*.

eFigure. Simplified Model Schematic for Connect for Health Markov Model Analysis.

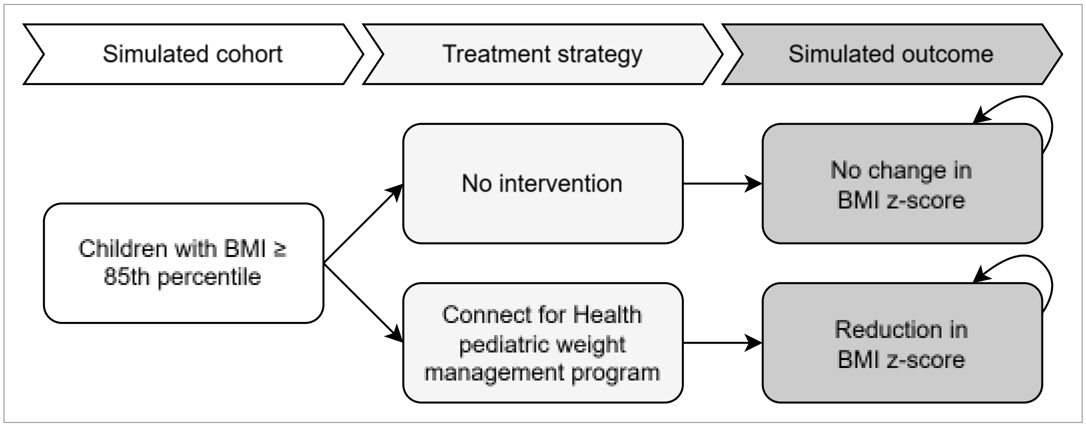

Note. *Abbreviations:* BMI, body mass index.

© 2025 Simione M et al. *JAMA Network Open*.

eMethods. Estimation of Overweight and Obesity Prevalence Among Children Aged 2-12 Served in Federally Qualified Health Centers.

Our objective was to estimate the number of Federally Qualified Health Centers (FQHCs) capable of implementing the Connect for Health program within the established willingness-to-pay threshold. For an FQHC to be considered viable for the program, it requires a minimum cohort size of 534 children with overweight or obesity aged 2-12 years, a threshold determined through the one-way sensitivity analysis. We used data from the 2022 UDS National Awardee report,[1] which detailed patient populations and income distributions at each FQHC. To estimate the number of children aged 2-12 at each facility, we referred to state-specific census data.[2] We assumed the income distribution in this age group mirrored that of the general population at each center. We then utilized NHANES 2017-2020 data[3] to estimate the prevalence of overweight and obesity among children aged 2-12 years, stratified by income. These prevalence rates were applied to the income-stratified patient populations to determine the expected number of overweight and obese children per FQHC. We concluded that a facility had a sufficient patient population for the program if it included a cohort of more than 534 children with overweight or obesity aged 2-12 years.

© 2025 Simione M et al. *JAMA Network Open*.

eReferences.

1. Health Resources & Services Administration (HRSA), U.S Department of Health & Human Services (HHS). National Health Center Program Uniform Data System (UDS) Awardee Data. Published online 2022. https://bphc.hrsa.gov/data-reporting/uds-training-and-technical-assistance/uds-data

2. U.S. Census Bureau. Annual Estimates of the Civilian Population by Single Year of Age and Sex for the United States and States: April 1, 2020 to July 1, 2023. Published online 2024. https://www.census.gov/data/tables/time-series/demo/popest/2020s-state-detail.html

3. Centers for Disease Control and Prevention (CDC), National Center for Health Statistics (NCHS). National Health and Nutrition Examination Survey Data 2017-March 2020 Pre-Pandemic. Published online 2021. https://wwwn.cdc.gov/nchs/nhanes/continuousnhanes/default.aspx?Cycle=2017-2020

© 2025 Simione M et al. *JAMA Network Open*.
