# Supplementary material for: Estimated Costs and Cost-Effectiveness of a Pediatric Weight Management Program
Source: JAMA Netw Open. 2025 May 14;8(5):e2510087. doi: 10.1001/jamanetworkopen.2025.10087 (PMC12079289; doi:10.1001/jamanetworkopen.2025.10087)
Supplement: Supplement 2. — Data Sharing Statement [file jamanetwopen-e2510087-s002.pdf]

Data Sharing Statement

Simione. Estimated Costs and Cost-Effectiveness of a Pediatric Weight Management Program. *JAMA Netw Open*. Published May 14, 2025. doi:10.1001/jamanetworkopen.2025.10087

Data
Data available: No

Additional Information
Explanation for why data not available: We will make the data available upon reasonable request.
